# Perceived injustice and its relation to chronic pain outcome in complex regional pain syndrome and chronic musculoskeletal pain

**DOI:** 10.3389/fpain.2025.1554630

**Published:** 2025-04-03

**Authors:** Corinna Baum, Cora Rebhorn, Anne Martinelli, Dorothee Heining, Sabine Weimert, Sandra Bücher, Livia Sophie Lang, Sebastian Steinmetz, Frank Birklein, Violeta Dimova

**Affiliations:** 1 Department of Psychology, Fresenius University of Applied Sciences, Frankfurt, Germany; 2 Department of Neurology, University Medical Center of the Johannes Gutenberg University, Mainz, Germany; 3 Department of Neuroradiology, University Medical Center of the Johannes Gutenberg University, Mainz, Germany

**Keywords:** complex regional pain syndrome, low back pain, chronic pain, perceived injustice, pain catastrophizing

## Abstract

**Objectives:**

Clinical observations indicate that patients with complex regional pain syndrome (CRPS) tend to ruminate about their illness. Perceived injustice is a negative cognitive-emotional appraisal regarding the severity of loss associated with blame, unfairness, and pain. We investigated injustice beliefs in CRPS compared with chronic musculoskeletal pain (CMP), where previous evidence indicates clinical relevance for pain-related outcome in this patients’ group. The role of perceived injustice in relation to pain intensity and disability was tested through a mediation model including catastrophizing thoughts of pain.

**Methods:**

Patients with CRPS (mean age *M* = 50.9, *SD* = 13.8) and CMP (mean age *M* = 53.9, *SD* = 8.0 years) were enrolled at two independent specialized outpatient clinics. All patients completed questionnaires on pain intensity, pain disability, and perceived injustice, levels of depression and pain catastrophizing.

**Results:**

CRPS patients displayed higher levels of perceived injustice than the CPM patients. Higher pain intensity in both cohorts was indirectly associated with more feelings and beliefs of injustice through a higher tendency to catastrophize about pain and pain-related information. In contrast, only in the CMP group higher pain-related disability was related to higher catastrophizing, which mediated the effect of perceived injustice.

**Conclusions:**

Perceived injustice influences especially pain intensity through pain catastrophizing. This interaction appears to be common for both pain syndromes.

## Introduction

Complex regional pain syndrome (CRPS) is a limb pain condition that usually develops after trauma or injury (1, 2). Along with severe pain, CRPS is characterized by sensory, autonomic, trophic and motor symptoms. While many CRPS cases improve within 6–12 months after standard treatment, a significant number of patients continue to experience severe pain and disability in the affected limb. As common in pain disorders, maladaptive psychological adjustment promotes the chronification of CRPS. Depression (3), prior psychological trauma and emotional distress (4), and kinesiophobia (5) have all been associated with CRPS persistence and related pain and disability. In particular, anxiety, pain-related fears and perceived disability have been shown to negatively predict 1-year disease outcome (6). Clinical observations indicate that patients with CRPS tend to ruminate about ‘why the injury happened to them’ and ‘what went wrong in healing’. In the case of limb pain after injury (7, 8) and after knee arthroplasty (9), such feelings of injustice have been associated with poor recovery outcomes. This is especially true for patients who have experienced a serious injury and have been hospitalized for an extended period of time, have received compensation for the injury, or have been unable to work for more than 12 months (8).

Perceived injustice defines a cognitive attitude characterized by negative appraisals regarding the severity of loss associated with blame, unfairness, and pain (10). It is associated with poor emotional and social functioning. Current evidence consistently supports a positive relationship between perceived injustice and different pain measures in chronic pain conditions (11). However, such a relationship has not yet been investigated in CRPS, which is remarkable because CRPS always has an external trigger event, which usually cannot be influenced by the patient. This might invite patients to engage in thoughts of injustice regarding pain. The available evidence mainly pertains to musculoskeletal pain and suggests a significant influence of injustice cognitions on pain intensity, disability, physical functioning and mental health (12). Mostly, injustice appraisal has been identified as a mediating cognition. The mediating role of anger has been demonstrated in the relationship between perceived injustice and pain intensity or disability as well (13).

In the present study, we therefore assessed levels of perceived injustice using the Injustice Experience Questionnaire (IEQ) in CRPS and compared the results to chronic musculoskeletal pain (CMP). CMP was chosen as a reference cohort since previous evidence indicates the clinical relevance of perceived injustice for pain-related outcome in this patient group. The role of perceived injustice in relation to pain intensity and disability was particularly tested by considering an indirect effect through catastrophizing thoughts of pain. That is, perceived injustice could be associated with higher pain catastrophizing, which in turn is known to have strong influences on pain perception (11).

## Methods

### Subjects and study design

Patients with CRPS or CMP were enrolled at two specialized outpatient clinics in Germany, namely the University Medical Center Mainz, and the Outpatient Rehabilitation Center Koblenz. All patients completed questionnaires on pain intensity, pain disability, and perceived injustice at a single assessment session. Additionally, levels of depression and pain catastrophizing were assessed according to the standardized diagnostic process in the clinical facility [e.g., Becks Depression Inventory (BDI) or Depression Anxiety Stress Scales (DASS), as well as the long or short forms of the Pain Catastrophizing Scale].

An a priori power analysis with G*Power for two-tailed correlations indicated that a minimum of 46 patients (per group) was required to detect a correlation of ρ = .40 between perceived injustice, pain catastrophizing and pain intensity and disability, with an alpha of 0.05 and a power of 80%. The underlying effect size was selected from two meta-analyses regarding pain catastrophizing (14) and perceived injustice (11) as predictors of affective and pain outcomes in (chronic) pain patients. The power analysis suggests that our groups with CRPS (n = 42) and CMP (n = 62) are suitable for the planned analyses.

### CRPS cohort

42 patients (mean age *M* = 50.9, *SD* = 13.8; 71.4% women; CRPS type 2 *n* = 5) diagnosed with CRPS of the upper or lower extremity were enrolled from the neurological outpatient clinic at the Department of Neurology of the University Medical Center in Mainz. Inclusion criteria were being at least 18 years of age and fulfillment of the Budapest research diagnostic criteria. Standardized neurologic examination included recording of the presence or absence of signs mentioned in the Budapest diagnostic criteria: pain signs (e.g., hyperalgesia, allodynia), autonomic changes such as temperature difference, changes in skin color, asymmetrical sweating and edema on the affected extremity, trophic changes like altered growing of nails and hairs, and motor signs (functional limitation and weakness) (15). The CRPS severity score (CSS) was calculated as a sum score of the presence of seven patient-reported symptoms and nine physician-confirmed signs (16).

### CMP cohort

62 patients with chronic musculoskeletal pain in the upper and lower back, joints, head and face (mean age *M* = 53.9, *SD* = 8.0 years; 64.5% women) were recruited from the Outpatient Rehabilitation Center Koblenz, which specializes in orthopedic pain treatment. Patients were recruited during their participation at a multidisciplinary pain management program. Inclusion criteria were being at least 18 years of age and a minimum of three months of pain symptoms as criterion for chronic pain (according to the International Association for the Study of Pain).

### Standard protocol approvals, registration and patient consents

The study followed the Declaration of Helsinki and was approved by the Ethics Committees of the Rhineland Palatinate Medical Association (No. 9142-F) and by the internal IRB of the Fresenius University of Applied Sciences, Frankfurt am Main, Germany. Informed written consent was obtained from each patient.

### Data acquisition

#### Acquisition of pain characteristics

The severity of chronic pain was assessed using the Chronic Pain Grade Questionnaire[CPG (17), German version (18)]. Pain intensity and disability were both measured using an 11-point numerical rating scale, where the pain intensity scores ranged from 0 = no pain to 10 = pain as bad could be. Pain disability is based on three items that assessed the extent of perceived disability in daily life, leisure activities, and work during the past three months. Two different scores could be calculated for pain-related disability: (a) the mean pain-related disability, which represented the mean score of the three items, multiplied by 100 and (b) the disability score (DS), which classified the disability mean value according to severity. The DS is calculated by assigning severity levels to the mean disability values: DS 0 ≤ 29, DS 1 = 30–49 =, DS 2 = 50–69, DS 3 ≥ 70 (19). The CPG showed good reliability of alpha = .82 (18).

#### Acquisition of psychological variables

The Injustice Experience Questionnaire [IEQ (20)], German version (21) assesses how individuals experience their present life influenced by injustice. Participants rate their experiences of 12 different thoughts, emotions and attitudes described in the questionnaire items, using a 5-point rating scale. In the present study, the mean value was used, ranging from 0 to 4, with high scores indicating a high degree of *perceived injustice*. The German IEQ showed good reliability (alpha = .93) (21).

*Pain catastrophizing* was measured using the Pain Catastrophizing Scale [PCS (22)], German version (23). The questionnaire asks for different thoughts and feelings that individuals may experience when they are in pain on a 5-point rating scale. The PCS yields a total score and three subscale scores assessing rumination, magnification and helplessness. The short form of the PCS (PCS-4), which consists of four items, was used in the CMP group. The PCS-4 has a good internal consistency and is equivalent to the original PCS (24). For analysis of pain catastrophizing levels across both patient groups, the mean scores of the PCS for both its original and the short form were utilized.

The *depression* level was assessed by the Becks Depression Inventory Revised [BDI-II (25), German version (26)] in CRPS patients and the Depression Anxiety Stress Scales [DASS (19)] in CMP patients. The BDI provides cut-off scores for mild (14–19), moderate (20–28) and severe depression (≥10). In the DASS values greater than 10 identify a suspected depression. Both questionnaires have showed good internal consistency [BDI-II: alpha = .92 (26); DASS-depression: alpha = .88 (19)].

### Data analysis

Statistical analyses were performed using SPSS Version 28.0.1.1 for Windows (IBM SPSS Inc., Chicago, USA) and JASP Version 0.17.1. Statistical significance was defined for all analyses as an alpha level of 0.05. Errors were normally distributed for all variables included in statistical analyses, which was tested by Q-Q Plots.

Group differences in demographic, pain, and psychological variables were analyzed using independent *t*-test, U-tests, or cross-tabulation statistics based on the respective scale (parametric vs. nonparametric). Analyses of variance (ANOVA) were conducted with the main factors ‘disease group’ and ‘pain duration’ on mean pain intensity, mean pain-related disability and the disability score. Pearson correlations were used to test intercorrelations among variables (i.e., pain variables and injustice, catastrophizing) in each patient group. Multiple regression analyses were conducted to identify predictors of pain intensity and pain-related disability. The main predictors included disease group, injustice, and catastrophizing along with the respective mean-centered interaction terms of group × injustice/catastrophizing. These interaction terms allowed to determine the potential interactive group-related predictive effect on the pain variables. As pain-related disability is highly influenced by pain intensity, the regression model controlled for pain intensity when predicting disability.

A bootstrapped mediation model was employed to investigate whether catastrophizing acted as a mediator between injustice (UV) and pain variables (DVs) in both cohorts. The indirect effect (mediation effect) was tested using percentile bootstrapping CI (95% confidence interval) with 5,000 resamples. This method performs best in small samples, i.e., increases the power of the analysis (27). The fit of different models was compared using the comparative fit index AIC (28), for instance, to assess whether the mediation model fit the prediction of pain intensity and disability equally well. The model with the minimum AIC is understood as the best fitting model.

## Results

### Demographic and clinical characteristics of the cohorts

There were no differences between the groups in terms of gender (*X*^2^ = 0.28, *p* = 0.600) or age (t = 1.23, *p* = 0.222). CRPS patients had significantly shorter pain duration (CRPS: M = 3.33, SD = 1.24; CMP: M = 5.02, SD = 1.19; U = 2,141.50, *p* < 0.001). This difference is due to the clinical indication of the recruitment centers (rehabilitation center vs. specialized outpatient clinic) and inclusion criteria (i.e., pain lasting for a minimum of 3 months in CMP patients). While pain intensity was not different between groups, CRPS patients reported higher levels of pain-related disability compared to CMP patients (t = −3.30, *p* = 0.001).

Within the CRPS cohort (*n* = 42, 71.4% female), pain duration was up to 6 months in 31.0% of patients and 6–12 months in a further 31.0%. The remaining patients suffer longer from pain (19.0% 12–24 months, 11.9% 24–60 months, and 7.1% over 60 months). Approximately half of the group reported surgery as the CRPS trigger (59.5%); 22.0% report a fracture and 14.3% a minor injury. In 66.7% of the patients the upper extremity and in 57.1% the right side were affected by CRPS. No difference was found in CRPS severity (CSS score) relating to affected extremity (upper: M = 10.6, SD = 3.6, *n* = 28; lower: M = 10.2, SD = 4.9, *n* = 14; t = 0.30, *p* = 0.769). On average, the CRPS group reached a mild level of depression (BDI: M = 16.3, SD = 11.4), 20% are categorized as mild and moderate level, and 10% as severe.

In the CMP cohort (*n* = 62, 64.5% female), pain was predominantly located in the (lower and upper) back (96.8%) and additionally in the head or facial region (14.5%) and the joints (40.3%). The majority of patients (51.6%) reported pain lasting for more than five years, while a smaller proportion reported pain lasting between 6 and 12 months (19.4%), 12–24 months (11.3%), or 24–60 months (17.7%). Of the patients, 40.3% reported a specific event that triggered their pain disorder including 48.0% who reported a trauma, 20.0% an illness, 16.0% a surgery, and 12.0% occupational disabilities. The CMP group displayed a low mean level of depression (DASS: M = 5.79; SD = 4.93). Fourteen patients (22.6%) reached or exceeded the cut-off of 10 for depression diagnosis. The demographic and clinical characteristics of the groups are outlined in Table 1.

**Table 1:** Descriptive and test statistics of disease group effects.

	CRPS (*N* = 42) M (SD)/*N* (%)	CMP (*N* = 62) M (SD)/*N* (%)
Demographic details		
Sex (% female)	71.4%	64.5%
Age	50.93 (13.79)	53.86 (8.02)
Pain duration (*n*)*		
<6 months	13 (31.0%)	0 (0.0%)
6–12 months	13 (31.0%)	12 (19.4%)
1–2 years	8 (19.0%)	7 (11.3%)
2–5 years	5 (11.9%)	11 (17.7%)
>5 years	3 (7.1%)	32 (51.6%)
CSS score	10.48 (4.01)	
Pain location (*n*)		
Upper extremities	28 (66.7%)	–
Back (lower and upper)	–	60 (96.8%)
Head and facial region	–	9 (14.5%)
Joints	–	25 (40.3%)
Pain metrics		
Pain intensity	61.83 (18.93)	58.71 (16.83)
Pain-related disability	68.02 (25.06)	52.04 (22.94)*
Disability score	1.83 (1.34)	1.66 (1.01)
Pain cognitions		
IEQ	1.71 (0.87)	1.29 (0.79)*
PCS	1.94 (1.01)	2.02 (1.00)

CRPS, complex regional pain syndrome; CMP, chronic musculoskeletal pain; IEQ, injustice experience questionnaire (max mean score = 4); PCS, pain catastrophizing scale (max mean score = 4); *statistically significant difference at *p* < 0.05 level.

### Between group comparisons in perceived injustice, pain catastrophizing and pain variables

The CRPS group reported significantly higher ratings of perceived injustice (t = −2.54, *p* = 0.013), while the groups showed no difference in pain catastrophizing (t = 0.38, *p* = 0.705) (Table 1). Neither levels of perceived injustice nor of pain catastrophizing and depression were significantly correlated with CSS scores [*r* = (0.04–0.17), *p* > 0.28]. ANOVAs showed no significant main effect of pain duration on pain intensity [F(4,95) = 0.42, *p* = 0.791], mean pain-related disability [F(4,95) = 0.31, *p* = 0.871] or the disability score [F(4,95) = 1.01, *p* = 0.404]. A significant main effect of group was found for mean pain-related disability [F(1,95) = 11.22, *p* = 0.001] but not for the disability score or pain intensity [disability score: F(1,95) = 0.61, *p* = 0.436; intensity: F(1,95) = 0.76, *p* = 0.385].

Pain intensity was positively correlated with catastrophizing in both disease groups (CRPS *r* = 0.481, *p* = 0.001; CMP *r* = 0.438, *p* < 0.001) and with injustice only in the CMP group (*r* = 0.341, *p* = 0.007). Furthermore, significant positive correlation coefficients were found between pain-related disability, and both injustice and catastrophizing in the CMP (*r* = 0.431, *p* < 0.001, *r* = 0.490, *p* < 0.001), but not in the CRPS group (*r* = 0.082, *p* = 0.601; *r* = 0.278, *p* = 0.075). Pain duration did not show any significant correlation (for coefficients, see Table 2).

**Table 2:** Correlations between injustice, catastrophizing, and pain variables.

	Injustice	Catastrophizing	Pain disability	Pain intensity
CRPS group				
Pain duration	0.141	0.153	0.141	0.119
Pain intensity	0.296	0.481**	0.693**	
Pain disability	0.083	0.278		
Catastrophizing	0.562**			
CMP group				
Pain duration	0.141	0.003	−0.107	−0.018
Pain intensity	0.341*	0.438**	0.594**	
Pain disability	0.431**	0.490**		
Catastrophizing	0.696**			

* *p* < 0.01, **p < 0.001.

### Predictors of pain intensity and pain-related disability

The results of the multiple regression analysis indicated that the overall model, which includes interaction terms, explained approximately 47% of the variance in pain-related disability [Adj *R*^2^ = 0.47, F(6,97) = 16.27, *p* < 0.001] (Table 3). Although both disease group and pain intensity significantly predicted pain-related disability, their interaction with injustice and pain catastrophizing did not reach statistical significance. This suggests that there is no significant interaction between disease group and the cognitive factors in predicting pain disability.

**Table 3:** Multiple regressions for prediction of pain disability and intensity.

	b (SE b)	ß	t	*p*
Predictors of pain-related disability				
Disease group	13.98 (3.88)	0.28	3.60	<0.001
Pain intensity	0.78 (0.11)	0.55	6.84	<0.001
Injustice	3.90 (4.10)	0.13	0.95	0.344
Catastrophizing	3.33 (3.34)	0.13	1.00	0.321
Injustice × disease group	−7.64 (5.70)	−0.17	−1.34	0.184
Catastrophizing × disease group	−1.69 (4.70)	−0.04	−0.36	0.720
Predictors of pain intensity				
Disease group	3.25 (3.41)	0.09	0.95	0.343
Injustice	1.51 (3.62)	0.07	0.42	0.678
Catastrophizing	6.56 (2.87)	0.37	2.29	0.024*
Injustice × disease group	−0.67 (5.03)	−0.02	−0.13	0.894
Catastrophizing × disease group	2.04 (4.15)	0.07	0.49	0.624

* *p* < 0.05.

The second regression model, which included disease group, catastrophizing, injustice and their interaction terms, explained 18% of the variance in pain intensity [Adj *R*^2^ = 0.17, F(5,98) = 5.45, *p* < 0.001]. The results indicate that higher levels of catastrophizing were significantly associated with higher pain intensity, after controlling for disease group.

### Mediation analysis: indirect effects of perceived injustice on pain intensity and disability

**Figure 1:**
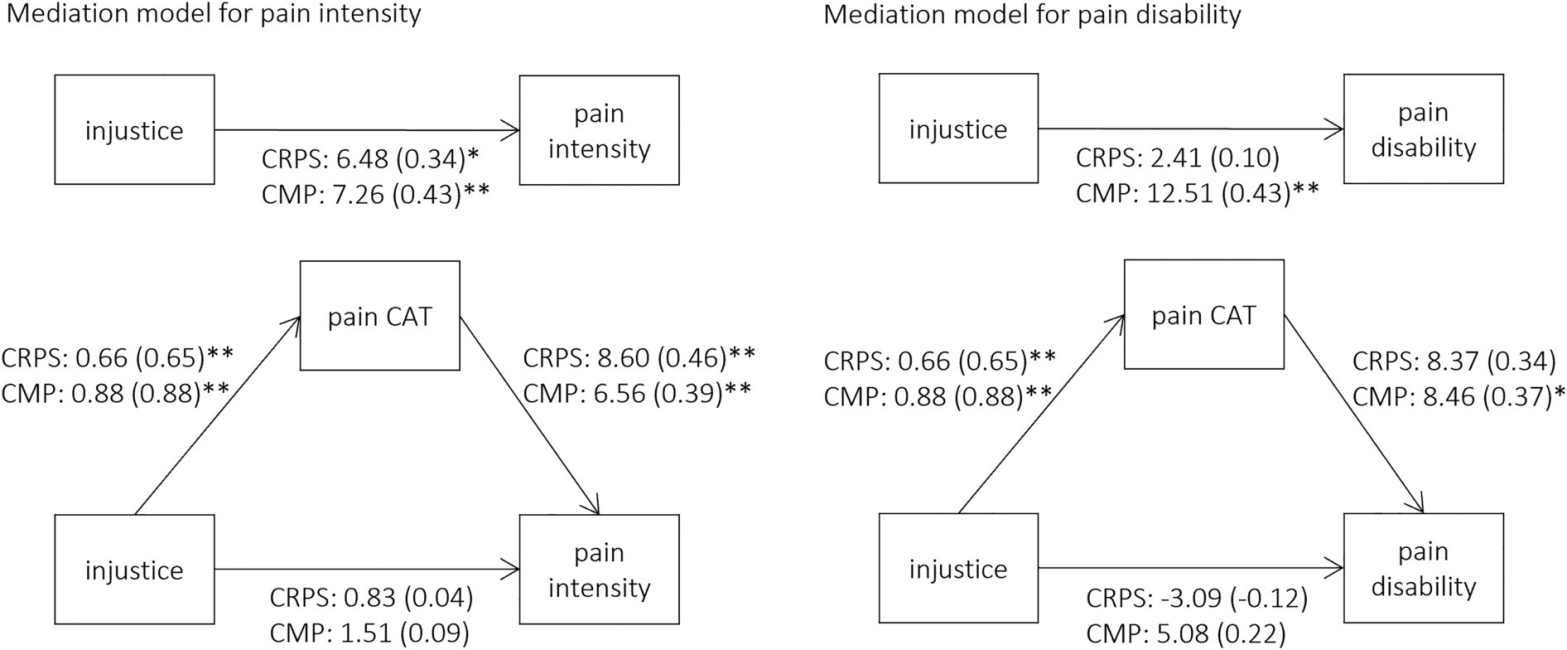
Pain catastrophizing mediation models of the relationship between injustice and pain variables for CRPS and CMP groups. Unstandardized coefficient (standardized coefficient), pain CAT = pain catastrophizing, * *p* < 0.05 ** *p* < 0.01.

Based on the covariation of injustice and catastrophizing, the indirect effect of injustice on pain intensity and pain-related disability mediated by catastrophizing was tested. We hypothesized that perceived injustice related to chronic pain diseases would directly increase pain-related catastrophizing and indirectly pain intensity and disability (see Figure 1). For CRPS, the mediator effect was significant for pain intensity (b = 5.65, *ß* = 0.30, se = 0.13, *p* = 0.018), but not for pain-related disability (b = 5.50, *ß* = 0.22, se = 0.13, *p* = 0.082). In contrast, for CMP, a significant mediator effect was found for both pain intensity (b = 5.76, *ß* = 0.34, se = 2.47, *p* = 0.020) and pain-related disability (b = 7.43, *ß* = 0.32, se = 2.23, *p* = 0.022).

Overall, for both CRPS and CMP, the mediation effect was stronger for pain intensity compared to pain-related disability, as supported by the comparative fit indices (CRPS: AIC-intensity = 581.26 vs. AIC-disability = 612.20, CMP: AIC-intensity = 808.88 vs. AIC-disability = 842.38).

## Discussion

Psychological factors are discussed as the most promising early predictors for chronic CRPS (29). Perceived injustice defines a cognitive attitude characterized by negative appraisals, specifically feelings of unfairness. It has been shown to influence the development of pain after injury, but studies on CRPS are lacking. We assessed levels of perceived injustice in persistent CRPS compared to chronic CMP, its association to pain catastrophizing cognitions and its mediating influence on pain experience.

CRPS patients displayed higher levels of perceived injustice than CMP patients. Higher pain intensity in both cohorts was directly associated with a tendency to catastrophize about pain and pain-related information, and indirectly associated with more feelings and beliefs of injustice. Only in the CMP group, higher pain-related disability was affected by higher pain catastrophizing, which mediated the influence of perceived injustice. Higher pain intensity in both cohorts was indirectly associated with more feelings and beliefs of injustice, mediated by an increased tendency to catastrophize about pain and pain-related information. Only in the CMP group, the mediation model was also confirmed for pain-related disability, which was affected by higher pain catastrophizing mediating the influence of perceived injustice.

### Elevated levels of perceived injustice in CRPS

CRPS patients showed high levels of perceived injustice. There are few studies suggesting the maintenance of longstanding CRPS by psychological factors, specifically anxiety and pain-related fear favoring avoidance behavior (6, 30). Elevated levels of depression were shown to associate with disability and sick leave in CRPS after severe injuries (3). Currently, no evidence exists regarding the role of injustice in CRPS. So far, perceived injustice was shown to mediate poor return to work after compensable traumatic injuries (not restricted to limb injuries) (7). In orthopaedic trauma, patients’ perceived injustice was correlated to pain intensity and physical functioning, however the stronger predictors were pain catastrophizing and pain-related self-efficacy (31). In contrast, in our CRPS cohort, perceived injustice was not correlated with either pain intensity or disability, but rather to pain catastrophizing. Similarly, in traumatic injury patients, the health impact of injury, catastrophizing and pain-related self-efficacy were stronger associated with injustice feelings than the injury or pain severity (8). In a large naturalistic pain clinic population, prevalence of 39%, 32% and 29% were reported for low, medium and high IEQ scores, respectively (32). The authors classified IEQ scores into low <19, medium 19–29 and high >30 levels of IEQ according to proposed clinical considerations (20, 33). Mean levels of IEQ in our CRPS cohort surpassed the medium cut-off score, whereby 29% and 26% of *n* = 42 CRPS patients displayed medium and high IEQ scores, respectively. In contrast, of the *n* = 62 CMP patients only 21% and 10% could be accordingly classified into medium and high IEQ levels. This suggests that the effects of injustice on pain outcomes in both disease groups might be based on different illness specific sources of injustice. In CRPS, feelings and cognition of injustice might be associated with the physical trauma and or the trauma related circumstances and their aversive effect rather than with the pain intensity itself. The strong experience of injustice related to the illness—as seen in the CRPS group—can also be understood more generally as (a part of) a negative illness perception. In a recent study, it was suggested for CRPS that this negative perception favors greater pain, disability and avoidance behavior (34). The authors interpret their results in the frame of Leventhal's common-sense model of self-regulation (35), where threat beliefs about the illness are generated by a cognitive illness perception (i.e., knowledge about the disease condition and impact on life as well as self-management beliefs) and emotional representation (i.e., emotional impact and suffering). It can be hypothesized that a corresponding cognitive-emotional approach governs perceived injustice and thus may play a crucial role in a maladaptive self-regulation under CRPS. In contrast, for CPM patients a direct link to pain could be suspected.

### Differential and common association between perceived injustice, pain outcome and pain catastrophizing in CRPS vs. CMP

In our study, pain catastrophizing was a direct predictor for pain intensity in both CRPS and CMP patients and furthermore could be identified to mediate the relationship between perceived injustice and pain intensity. For pain disability, the direct or mediating effect of catastrophizing in a complete model considering perceived injustice fit only for CMP patients.

First, these effects confirm the fundamentally strong association between pronounced catastrophic appraisal of pain and increased pain intensity, regardless of diagnostic and pain-specific aspects. Accordingly, perceived injustice seems to play a rather indirect role in reported pain intensity when catastrophizing is taken into account. In principle, the relationship between catastrophizing and intensity may also be circular in nature or at least correspond to a strong covariation, with catastrophizing being additionally triggered by perceived injustice, as further studies confirm (10).

Regarding levels of disability, this appears to be best predicted by higher pain intensity itself and the presence of a specific chronic pain disorder. For example, CRPS patients reported higher pain disability compared to CMP, which explained a significant amount of variance in disability. While cognitive aspects of pain appraisal are typically highly associated with pain disability (20), they assume a secondary role when considering individual pain intensity. This seems logical, as disability determination is directly tied to the degree of limitation caused by experienced pain across various life domains. Therefore, disability tends to be more of a direct reaction to pain (intensity) rather than primarily driven by cognitions related to pain. If disability would more strongly represent avoidant behavior due to pain-related cognitions and emotions, the influence of individual styles in pain cognitions could emerge as more prominent. This may explain why some recent studies confirmed a direct effect of perceived injustice on pain-related outcomes, whereby stronger relations were found for pain-related disability compared to pain intensity (20). However, possible mediation effects of pain-related appraisal styles e.g., catastrophizing, were not considered. First evidence for the reduction of the predictive value of perceived injustice on pain disability and intensity through additional consideration of catastrophizing could be found by Ljosaa and colleagues (32). The present study confirmed that the predictive value of catastrophizing exceeded that of perceived injustice. Notwithstanding, both predictors demonstrated a relevant overlap, which could from a theoretical point of view either be based on shared content of the concepts or on common activation of pain-related cognitions or coping behavior. Especially the issue of (irreparability of) loss is interpreted as common in both concepts (and questionnaires). On the other hand, a dispositional style of injustice perception and catastrophizing thinking might both activate concepts of (learned) helplessness/hopelessness, which in turn could refer to a dysfunctional pain coping behavior of avoidance and disuse. These maladaptive behaviors in turn are discussed to affect chronic pain and further the entire process of pain chronification.

### Limitations

The two cohorts were recruited from different inpatient centers (a neurologic clinic with primary medical treatment indications vs. a rehabilitation center) with originally independent study designs. This produced mainly differences in the disease duration of the patients, with the CPM group being more homogeneous than the CRPS group with patients in the post-acute and chronic phase. Additionally, different measures for depression and pain catastrophizing were used. As this is a cross-sectional study, the examination of the relationship between injustice and pain outcomes is correlational. Providing evidence for a reciprocal relationship between injustice and catastrophizing requires a longitudinal design. However, it is also conceivable that pain and disability grow along with disease duration or chronicity while the level of injustice, being a strongly trait-associated variable, remains stable. Lastly, it should be noted that pain catastrophizing is just one potential pathway through which injustice affects pain outcomes. Additional affective, cognitive, and behavioral variables such as pain behavior or pain acceptance may serve as additive or interacting mediators with catastrophizing.

## Conclusions

Perceived injustice acts indirectly in both groups, especially on pain intensity through the influence of pain catastrophizing. Future studies involving additional patient groups are necessary to further develop the model of the indirect significance of injustice.

## Data Availability

The raw data supporting the conclusions of this article will be made available by the authors, without undue reservation.
